# Association of individual and bundled evidence-based practices with adverse outcomes in very preterm infants: a multicenter retrospective study

**DOI:** 10.3389/fped.2026.1785689

**Published:** 2026-05-20

**Authors:** Jing Feng, Xin Guo, Rui Li, Haiping Cheng, Yuanyuan Li, Kanghua Zhou, Dandan Rao, Guilian Du, Xudong Yan, Zhangbin Yu, Cheng Chen, Huiying Tu

**Affiliations:** 1 Department of Neonatology, Longgang District Maternity & Child Healthcare Hospital of Shenzhen City (Longgang Maternity and Child Institute of Shantou University Medical College), Shenzhen, China; 2 Department of Neonatology, The People’s Hospital of Baoan Shenzhen, Shenzhen, China; 3 Department of Neonatology, Shenzhen Baoan Women’s and Children’s Hospital, Shenzhen, China; 4 Department of Neonatology, Shenzhen Luohu People’s Hospital, Shenzhen, China; 5 Department of Neonatology, Shenzhen Bao'an District Songgang People’s Hospital, Shenzhen, China; 6 Department of Neonatology, Shenzhen People’s Hospital, the Second Clinical Medical College, Jinan University, The First Affiliated Hospital, Southern University of Science and Technology, Shenzhen, China

**Keywords:** evidence-based practices, mortality, outcome, survival without major morbidity, very preterm infants

## Abstract

**Background:**

Although evidence-based practices (EBPs) can improve outcomes in very preterm infants (VPIs), real-world implementation rates and evidence regarding their synergistic effects remain limited. This study, which is based on the Shenzhen Neonatal Data Network (SNDN), evaluated the current implementation status of EBPs and the impact of their synergistic effects on clinical outcomes.

**Methods:**

We conducted a retrospective study on 536 VPIs with a gestational age <32 weeks in 2024. These infants were categorized into a survival without major morbidity group and an adverse outcome group (severe complications/death), with differences in the implementation of EBPs evaluated between the two groups. Using the “all-or-none” approach, we further assessed four core EBPs: complete course of antenatal corticosteroids (ACS); antenatal magnesium sulfate (MgSO₄); maintenance of normothermia (36.5 °C–37.5 °C) within one hour after birth; and caffeine therapy. Multivariate logistic regression analysis was employed to explore the associations between EBP implementation and adverse outcomes.

**Results:**

Bundle implementation of four EBPs significantly reduced adverse outcomes in VPIs (aOR=0.44, 95% CI: 0.23–0.83). Independent protective factors included delivery at tertiary perinatal centers with RDS management capabilities (aOR=0.18, 95% CI: 0.04–0.86); and normothermia within the first hour after birth (aOR=0.58, 95% CI: 0.37–0.91). Gestational age stratified analysis revealed that the protective effect of the four EBPs bundle was most pronounced in infants <28 weeks (aOR=0.22, 95% CI: 0.06–0.78).Only 19.4% of VPIs received all four EBPs.

**Conclusions:**

Bundled implementation of EBPs significantly improves clinical outcomes in VPIs. However, suboptimal adherence—particularly for normothermia within the first hour after birth—necessitates targeted quality improvement initiatives. We can enhance quality and improve survival without major morbidity in VPIs by systematically improving the implementation rates of EBPs.

## Introduction

Preterm birth is the leading cause of neonatal mortality and is associated with long-term physical, neurodevelopmental, and socioeconomic effects, especially for very preterm infants (VPIs) (1). Although improvements in neonatal care have increased survival without major morbidity among VPIs (2, 3), the burdens of neonatal mortality and severe complications attributable to VPIs remain substantial (4).

The implementation of evidence-based practices (EBPs) can reduce preterm infant mortality, effectively prevent severe complications, and improve clinical outcomes (5–7). Although evidence-based guidelines have been established in various countries, significant variations exist in the implementation of these practices. Systematic research concerning the actual implementation rates in real-world clinical settings and the cumulative impact on preterm infant outcomes is lacking.

This study retrospectively evaluated the implementation of EBPs for VPIs with a gestational age (GA) < 32 weeks in the Shenzhen Neonatal Data Network(SNDN) on the basis of the 2022 European Consensus Guidelines for Respiratory Distress Syndrome (RDS) management (8). The cumulative effects of four key EBPs on clinical outcomes in this population were further analysed, aiming to provide evidence for standardized clinical practice.

## Patients and methods

### Study population

Inclusion criteria: Data for this multicenter study were obtained from the Shenzhen Neonatal Data Network databases on VPIs with a GA <32 weeks who were admitted to the neonatal intensive care unit (NICU) between 1st January 2024 and 31st December 2024.

**Figure 1:**
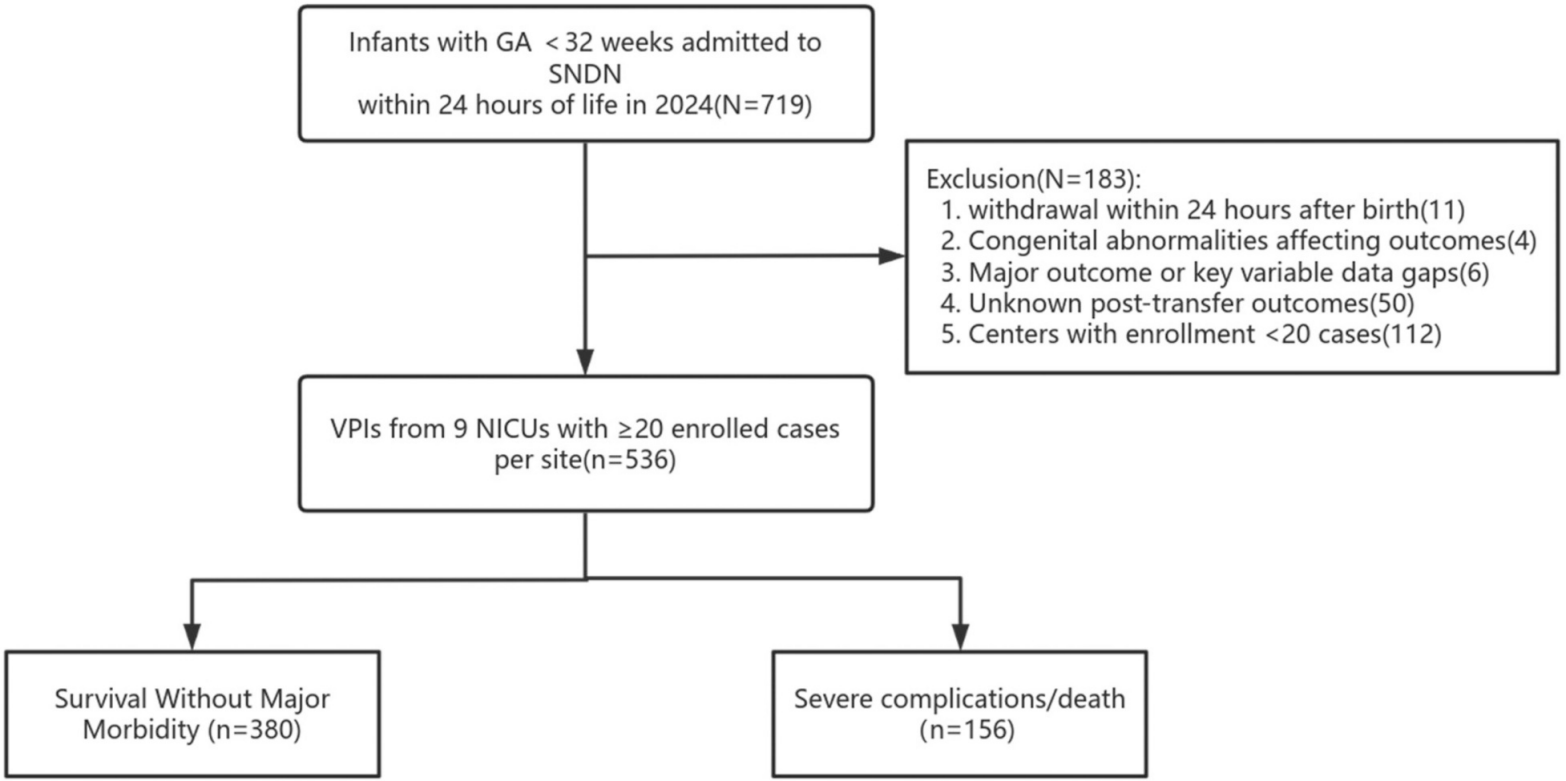
Patient enrollment flowchart.

The exclusion criteria were as follows: (1) treatment withdrawal for nonmedical factors within 24 h after birth (*n* = 11); (2) major congenital anomalies that independently determine poor prognosis and may interfere with the assessment of EBP effects (*n* = 4); (3) major data gaps (*n* = 6); (4) the outcome after transfer treatment is unknown (*n* = 50); and (5) patients from centers with enrollment <20 (*n* = 112) (Figure 1).

### Definition of EBPs: using an all-or-none approach

We used an all-or-none approach to study the use of EBPs. In contrast with an item-by-item assessment of performance or the creation of a composite measure, this approach considers whether all measures have been provided to each eligible patient (9).

A restrained set of indicators is selected that should measure performance on the specified elements of good practice and be related to the desired outcomes. Our study evaluated adherence to the 10 EBPs outlined in the protocol of the 2022 European respiratory distress syndrome guidelines, from which we identified four with a high level of evidence related to neonatal mortality and morbidity that could be measured reliably via information from medical records. Some EBPs included in the protocol were excluded because of inconsistent implementation by all hospitals, such as deferring cord clamping (DCC), umbilical cord milking (UCM), and the use of a T-piece resuscitator.Inborn delivery was not included in the bundle analysis due to near-universal implementation.

The selected indicators were (1) a complete course of antenatal corticosteroids (ACSs) before delivery; (2) antenatal magnesium sulfate (MgSO_4_); (3) normothermia (36.5 °C‒37.5 °C) within the first hour after birth; and (4) caffeine therapy.

### Definition of outcomes

The primary outcome was a composite of severe complications and/or death. Severe complications were defined as the presence of any of the following: ≥grade 3 intraventricular hemorrhage (10) and/or periventricular leukomalacia (IVH/PVL) (11), moderate-to-severe bronchopulmonary dysplasia (BPD) (defined by the NICHD 2001 criteria) (12), grade 3 necrotizing enterocolitis (NEC) (13), and ≥ grade 3 retinopathy of prematurity (ROP) and/or surgical treatment (14). Death was defined as either in-hospital mortality or death within 24 h after treatment withdrawal. Survival without major morbidity is defined as survival to 36 weeks postmenstrual age or hospital discharge (whichever comes first) without grade ≥3 IVH/PVL, Bell's stage ≥3 NEC, moderate/severe BPD, or severe ROP.

### Covariables

We identified clinical and healthcare factors likely to influence the probability of receiving evidence-based practices and outcomes. These factors included gestational age, sex, birth weight, small for gestational age, type of delivery, multiple pregnancy, maternal age, gestational diabetes mellitus (GDM), hypertensive disorders of pregnancy (HDP), assisted reproductive technology (ART), and 1-minute and 5-minute Apgar scores.

### Statistical analyses

All continuous variables were subjected to normality assessment via the Shapiro‒Wilk test. Nonnormally distributed continuous variables are summarized as medians (interquartile ranges, IQRs) and were compared with the Mann‒Whitney U test. Categorical variables are described as frequencies and percentages and were analysed by the chi-square test. Missing data (<5%) were handled by complete-case analysis. The impact of EBP measures on adverse outcomes was assessed via logistic regression analysis. A multivariate logistic regression model was used to adjust for potential confounding factors, including gestational age, sex, birth weight, delivery mode, conception method, and a 1-minute Apgar score ≤7. Adjusted results are reported with adjusted odds ratios (aORs) and 95% confidence intervals (95% CIs). All the statistical analyses were conducted via SPSS Statistics 27.0, with a *p* value <0.05 considered statistically significant.

## Results

### Study population characteristics

A total of 536 VPIs with a GA < 32 weeks were included, with an overall median GA of 30.0 weeks and a median birth weight (BW) of 1,245 g. These infants were stratified into survival without major morbidity (*n* = 380) and severe complication/death (*n* = 156) groups. Significant intergroup differences were observed: the severe complications/death group had lower GA (median 27.9 vs. 30.4 weeks, *P* < 0.001) and BW (median 960 g vs. 1,360 g, *P* < 0.001) values. Higher rates of assisted reproductive technology (26.3% vs. 18.0%, *P* = 0.031), lower cesarean section rates (59.6% vs. 73.7%, *P* = 0.001), and a higher proportion of patients with a 1-minute Apgar score ≤7 (30.1% vs. 13.7%, *P* < 0.001) were observed in the severe complications/death group. Sex, maternal age, hypertensive disorders, and a 5-minute Apgar score ≤7 were not significantly different (*P* > 0.05) (Table 1).

**Table 1:** Baseline characteristics and outcomes.

Variables, n(%)	survival without major morbidity (*n* = 380)	Severe complications/death (*n* = 156)	Total (*n* = 536)	*χ*^2^/Z	*P* value
Gestational Age(weeks)median (IQR)	30.4 (29.1,31.3)	27.9 (26.1,29.7)	30 (28.1,31)	−10.574	<0.001*
Sex, Male	212 (55.8)	101 (64.7)	313 (58.4)	3.65	0.056
Birth Weight(g), median(IQR)	1,360 (1,130,1,520)	960 (800,1,200)	1,245 (990,1,490)	−10.294	<0.001*
small for gestational age	17 (4.5)	12 (7.7)	29 (5.4)	2.239	0.135
ART	68 (18)	41 (26.3)	109 (20.4)	4.674	0.031*
multiple pregnancy	105 (27.7)	46 (29.5)	151 (28.2)	0.641	0.726
maternal age(y), median(IQR)	32 (29,35)	31 (29,35)	31 (29,35)	−0.795	0.426
Cesarean section	278 (73.7)	93 (59.6)	371 (69.6)	10.405	0.001*
GDM	127 (33.4)	39 (25.2)	166 (31)	3.51	0.061
HDP	75 (19.8)	33 (21.3)	108 (20.3)	0.143	0.705
1-minute Apgar score<7	52 (13.7)	47 (30.1)	99 (18.5)	19.86	<0.001*
5-minute Apgar score<7	11 (2.9)	9(5.8)	20(3.7)	6.275	0.111

GDM, gestational diabetes mellitus; HDP, hypertensive disorders of pregnancy; ART, assisted reproductive technology.

*p < 0.05 indicates statistical significance.

To assess potential selection bias introduced by excluding infants from smaller centers (*n* = 112) and those transferred with unknown outcomes (*n* = 50), we compared the baseline characteristics of included (*n* = 536) and excluded (*n* = 162) very preterm infants (Supplementary Table S1). The two groups were largely comparable in terms of gestational age, birth weight, proportions of small-for-gestational-age, multiple pregnancy, maternal age, cesarean section, gestational diabetes, hypertensive disorders, and Apgar scores (all *P* > 0.05). However, the excluded group had a significantly lower proportion of males (46.9% vs. 58.4%, *P* = 0.010) and lower use of assisted reproductive technology (11.9% vs. 20.4%, *P* = 0.015).

### Evidence-Based practices (EBPs) adherence

Adherence to EBPs was significantly different between the two groups. The severe complications/death group exhibited significantly lower adherence to key EBPs (Table 2): complete ACS (44.9% vs. 55.8%, *P* = 0.021). DCC/UCM (4.9% vs. 18.5%, *P* = 0.020); normothermia (36.5 °C-37.5 °C) within the first hour after birth (36.5% vs. 54.7%, *P* < 0.001); caffeine administration rate within 48 h after birth (88.3% vs. 94.3%, *P* = 0.024); and enteral nutrition within 24 h (42.9% vs. 67.0%, *P* < 0.001). T-piece resuscitator use was greater in the severe complications/death group (75.8% vs. 65.8%, *P* = 0.008), likely reflecting greater illness severity.

**Table 2:** Outcome comparisons by exposure to specific evidence-based practices (EBPs).

EBPs	survival without major morbidity(*n* = 380)	Severe complications/death(*n* = 156)	Total (*n* = 536)	*Χ*^2^/Z	*P* value
ACS	337 (89.6)	141 (91.6)	478 (90.2)	0.46	0.497
Complete course of ACS	212 (55.8)	70 (44.9)	282 (52.6)	5.288	0.021*
Magnesium sulfate	282 (74.2)	107 (68.6)	389 (72.6)	1.755	0.185
Inborn delivery	376 (98.9)	150 (96.2)	526 (98.1)	-	0.04*
DCC/UCM	99 (18.5)	26 (4.9)	125 (23.3)	5.449	0.02*
T-piece use in the delivery room	235 (65.8)	113 (75.8)	348 (68.8)	15.027	<0.001*
Normothermia	208 (54.7)	57 (36.5)	265 (49.4)	14.653	<0.001*
PS	165 (43.4)	119 (76.3)	284 (53)	47.942	<0.001*
Initial dose of PS	173.9 (142.9,200)	179.8 (142.4,200)	176.5 (142.9,200)	−1.287	0.198
Caffeine therapy	335 (88.2)	137 (87.8)	472 (88.1)	0.012	0.913
Caffeine within 48 h after birth	316 (94.3)	121 (88.3)	437 (92.6)	5.111	0.024*
Enteral feeding within 24 h	238 (67)	57(42.9)	295(60.5)	23.671	<0.001*

ACS, antenatal corticosteroids; DCC, deferring cord clamping; UCM, umbilical cord milking; PS, pulmonary surfactant.

*p < 0.05 indicates a statistically significant difference.

**Figure 2:**
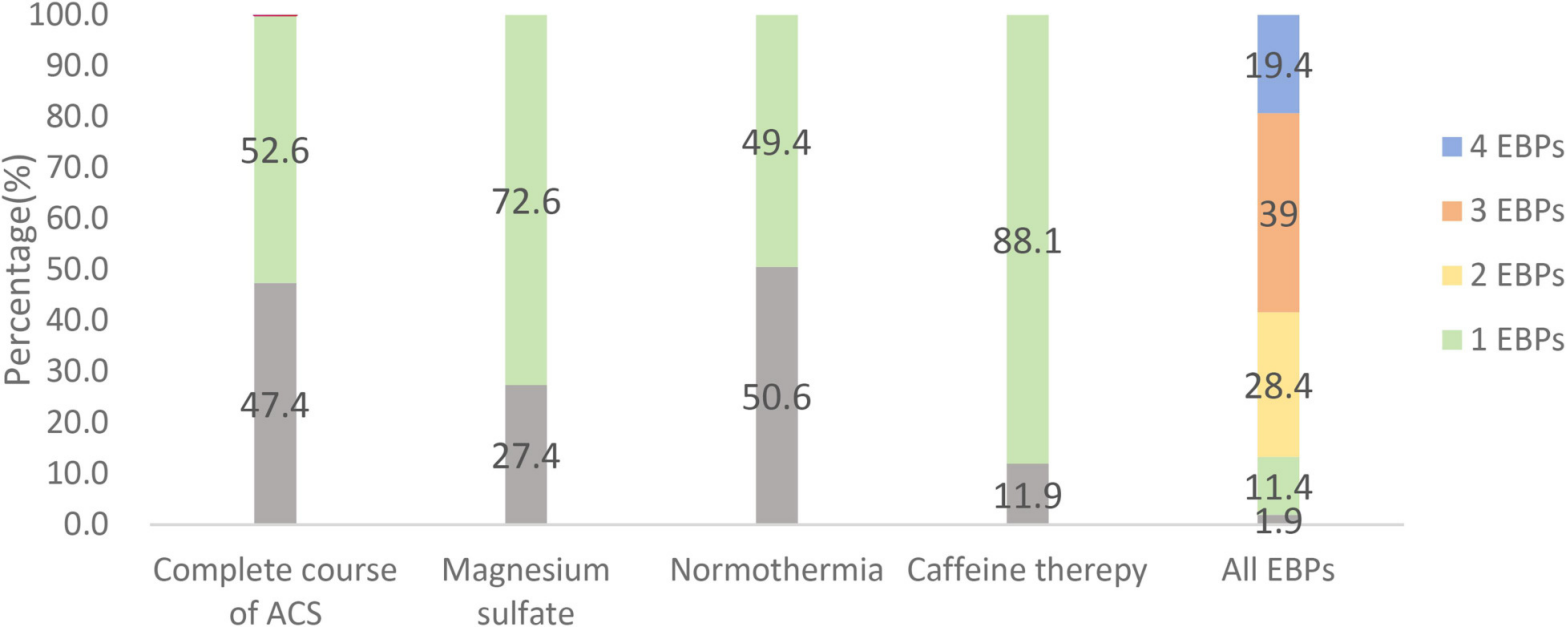
All-or-none adherence rate to EBPs.

 Figure 2 shows the implementation of all or none of the four EBPs. Specifically, 52.6% (*n* = 282) received a complete course of ACS, 72.6% (*n* = 389) were administered antenatal MgSO_4_ for fetal neuroprotection, 49.4% (*n* = 265) achieved normothermia (36.5–37.5 °C) within the first hour after birth, and 88.1% (*n* = 472) received caffeine therapy. However, only 19.4% (*n* = 104) of the infants received all four EBPs concurrently (Figure 2).

### Impact of EBPs on outcomes

Multivariate regression analysis (adjusted for gestational age, sex, birth weight, conception method, delivery mode, and 1-minute Apgar score ≤7) revealed that three EBPs were significantly associated with a reduction in severe complications/deaths: delivery at tertiary perinatal centers with RDS management capabilities(aOR=0.18, 95% CI: 0.04–0.86; *P* = 0.032). DCC/UCM (aOR=0.55, 95% CI: 0.31–0.96, *P* = 0.034), and normothermia (36.5–37.5 °C) within the first hour after birth (aOR=0.58, 95% CI: 0.37–0.91, *P* = 0.019) (Table 3).

**Table 3:** Logistic regression analyses examining the association of EBPs with severe complications/death.

Variables	Crude OR(95%CI)	*P* value	Adjusted OR(95%CI)	*P* value
Complete course of ACS	0.65 (0.44,0.94)	0.022	0.71 (0.45,1.12)	0.139
Magnesium sulfate	0.76 (0.50,1.14)	0.186	0.61 (0.37,1.0)	0.051
Inborn delivery	0.27 (0.07,0.96)	0.042	0.18 (0.04,0.86)	0.032*
DCC/UCM	0.57 (0.35,0.92)	0.021	0.55 (0.31,0.96)	0.034*
Normothermia	0.48 (0.32,0.70)	<0.001	0.58 (0.37,0.91)	0.019*
Caffeine therapy	0.97 (0.55,1.72)	0.913	1.06 (0.51,2.22)	0.88
Bundled EBPs	0.39 (0.22,0.69)	0.001	0.44 (0.23,0.83)	0.011*

OR, odds ratio; CI, confidence interval.

Adjusted for gestational age, sex, birth weight, mode of conception, type of delivery, and 1-min Apgar score.

Bundled EBPs: Complete course of ACS; Magnesium sulfate; Normothermia and caffeine therapy.

*p < 0.05 indicates a statistically significant difference.

 Although a complete course of ACS, antenatal MgSO_4_, and caffeine therapy demonstrated no independent protective effects after adjustment (*P* > 0.05), concurrent implementation of all four EBPs significantly reduced adverse outcome risk (aOR=0.44, 95% CI: 0.23–0.83, *P* = 0.011) (Table 3).

To evaluate the impact of evidence-based practices (EBPs) on clinical outcomes across different gestational ages, we performed a stratified analysis by gestational age (<28 weeks vs. 28–31⁺⁶ weeks). As shown in Table 4, the results showed that among extremely preterm infants (<28 weeks), receipt of all four EBPs (complete course of antenatal corticosteroids, magnesium sulfate, normothermia maintenance, and caffeine therapy) was associated with a significantly reduced risk of adverse outcomes (adjusted OR = 0.22, 95% CI: 0.06–0.78, *P* = 0.02). In preterm infants at 28–31⁺⁶ weeks, although a protective trend was observed, it did not reach statistical significance (adjusted OR = 0.59, 95% CI: 0.28–1.23, *P* = 0.158). For individual EBPs, although magnesium sulfate (adjusted OR = 0.36, *P* = 0.095) and normothermia maintenance (adjusted OR = 0.43, *P* = 0.089) showed some protective trends in the <28 weeks group, none reached statistical significance.

**Table 4:** Effect of Evidence-Based Practices on Clinical Outcomes by Gestational Age.

Variables	<28w		28–31 ^+^ ^6^w	
	Adjusted OR(95%CI)	*P* value	Adjusted OR(95%CI)	*P* value
Complete course of ACS	1.04 (0.39,2.78)	0.939	0.92 (0.51,1.65)	0.767
Magnesium sulfate	0.36 (0.11,1.19)	0.095	0.69 (0.39,1.24)	0.217
Normothermia	0.43 (0.16,1.14)	0.089	0.64 (0.38,1.1)	0.107
Caffeine therapy	1.71 (0.41,7.16)	0.463	1.05 (0.43,2.58)	0.908
Bundled EBPs	0.22 (0.06,0.78)	0.02*	0.59 (0.28,1.23)	0.158

OR, odds ratio; CI, confidence interval.

Adjusted for gestational age, sex, birth weight, mode of conception, type of delivery, and 1-min Apgar score.

Bundled EBPs: Complete course of ACS; Magnesium sulfate; Normothermia and caffeine therapy.

*p < 0.05 indicates a statistically significant difference.

## Discussion

The all-or-none assessment method enables effective evaluation of the implementation status and improvement opportunities for high-quality EBPs with extensive validation (5, 15–17). In this study, we applied the all-or-none approach to analyse the four EBPs. Our analysis demonstrated that VPIs receiving all EBPs presented a significantly reduced incidence of adverse outcomes. These findings indicate that the comprehensive implementation of high-quality evidence-based guidelines improves preterm infant prognosis and reduces adverse event rates. However, only 19.4% received the complete bundle, indicating significant gaps in quality improvement—particularly for underperforming interventions such as normothermia within the first hour after birth (49.4%) and DCC/UCM (23.3%), which demonstrated critically low implementation rates in this study.

 The four EBPs bundled in our study have each been proven to improve the clinical outcomes of VPIs. For the first practice (complete course of ACS), meta-analyses of observational studies have shown that ACS reduces the risk of perinatal death. neonatal death, and probably reduces the risk of IVH. The administration rate for infants born at <32 weeks ranges from 82% to 93% in developed countries (18). In China, a large-scale survey reported an ACS utilization rate of 78.0%, with only 49.1% of these cases receiving a complete course (19). Our study revealed higher rates: 90.2% for ACS administration and 52.6% for the complete course. These figures exceed China's national averages and approach those documented in developed countries. The dosage, treatment course completeness, and timing of ACS administration may all potentially impact outcomes in VPIs (20–23). In our study, although the group receiving a complete course of ACS demonstrated a significantly lower incidence of adverse outcomes than the survival without major morbidity group did, the aOR was not significantly different. This may be attributed to our composite outcome incorporating multiple severe complications, which potentially weakened the association between ACS exposure and individual adverse outcomes. For the second EBPs (antenatal MgSO_4_), substantial evidence confirms that antenatal MgSO_4_ administration reduces the risk of cerebral palsy, although it has no significant association with overall infant mortality (24, 25). Although not associated with short-term clinical outcomes, MgSO₄ remains recommended as standard practice because of its established association with significantly reduced long-term neurodevelopmental sequelae such as cerebral palsy. Similarly, in our study, MgSO₄ was not significantly associated with short-term outcomes. However, when evaluated through the all-or-none composite assessment, it contributes to improved preterm infant outcomes when it is implemented collectively with other evidence-based interventions. MgSO_4_ administration increased from 65.8% in 2017 to 85.5% in 2022 in England (26). The administration rate of MgSO4 in our cohort suggests that there remains substantial room for improvement, consistent with findings from other real-world settings. Our third evidence-based practice targeted the maintenance of normothermia within the first hour after birth. A recent systematic review published in Pediatrics demonstrated that the mean hypothermia rate was 42% (range 14%–88%), and hypothermia was associated with increased mortality (27). In line with previous reports, our study found that maintaining normothermia within the first hour after birth was associated with a significantly lower risk of adverse outcomes. However, the observed incidence rate of hypothermia (50.6%) remained higher than the benchmark rates, necessitating targeted improvement initiatives. For the fourth evidence-based practice (caffeine therapy), meta-analyses indicate that caffeine administration reduces the incidence of BPD (28) and decreases the risk of extubation failure (29). However, the optimal timing for caffeine initiation remains debated (30). In our cohort, caffeine administration within 48 h after birth was significantly less frequent in the group with adverse outcomes, suggesting the potential importance of timely caffeine therapy.

DCC is another evidence-based practice that synergistically reduces neonatal mortality when combined with other EBPs (16). A meta-analysis provided high-certainty evidence that DCC reduces death before discharge in preterm infants (31). Similarly, our study found that DCC or UCM was associated with a reduced risk of adverse outcomes, consistent with previous meta-analyses. However, its limited sample size precluded inclusion in the bundle analysis.

The stratified analysis by gestational age in this study suggests that the comprehensive implementation of all four EBPs has a significant protective effect on extremely preterm infants <28 weeks, but does not reach statistical significance in preterm infants at 28–31⁺⁶ weeks. This difference may reflect the greater dependency of extremely preterm infants on bundled interventions, resulting in more pronounced benefits from EBP implementation. Future studies should further expand sample sizes to evaluate optimal strategies for EBP combinations across different gestational ages.

Translating these EBPs into routine practice requires attention to both feasibility and cost. Fortunately, all are relatively low-cost interventions: ACS and MgSO₄ are inexpensive medications, caffeine is generic, and normothermia management relies on basic thermal care practices. Nevertheless, real world adoption faces several barriers: the need for timely multidisciplinary collaboration between obstetrics and neonatology, adequate equipment and training for consistent temperature monitoring. The wide variation in implementation rates observed in our network (ranging from 49% to 88%) clearly reflects these challenges—and underscores that substantial room for improvement remains in real world clinical practice. Future research should apply implementation science frameworks to identify context-specific strategies and evaluate the cost-effectiveness of bundled implementation, thereby supporting targeted quality improvement initiatives.

There are several limitations in this study. First, we included only facilities with >20 VPI cases in the Network database. All nine sites were tertiary hospitals, limiting the generalizability of the conclusions to primary care settings. Second, the exclusion of infants from smaller centers and those transferred with unknown outcomes may have introduced selection bias. Although baseline characteristics showed that key prognostic indicators remained comparable between included and excluded groups, residual confounding cannot be entirely ruled out, which may limit the generalizability of our findings.Third, the retrospective design resulted in a reliance on medical record completeness for certain EBPs. For example, DCC implementation rates may be artificially low because of potential underreporting. Fourth, our study population was limited in terms of short-term outcomes up to hospital discharge, precluding analysis of long-term neurodevelopmental outcomes.Fifth, infants with major congenital anomalies were excluded because such conditions independently determine prognosis and may obscure the association between EBPs and outcomes. This limits the generalizability of our findings to this subpopulation, warranting future studies focusing on the role of EBPs in these vulnerable infants. Sixth, for antenatal interventions such as ACS and MgSO₄, the lack of administration may in some cases be attributable to imminent delivery due to dire intrauterine conditions (for example, placental abruption, cord prolapse, or severe fetal distress), which themselves are associated with poor neonatal outcomes. This potential indication bias could not be fully adjusted for in our retrospective analysis, as we lacked detailed data on the specific reasons for non-administration. Consequently, the observed protective effects of these interventions may be underestimated.

In summary, we demonstrated that bundle implementation of EBPs significantly reduces adverse outcomes in VPIs. Adherence to these practices can serve as a metric for evaluating a healthcare facility's quality of care and provide actionable insights for quality improvement. Nevertheless, the overall implementation rate of EBPs remains suboptimal, highlighting persistent challenges in translating effective interventions into routine clinical practice. Barriers include gaps in clinicians’ knowledge and attitudes, organizational obstacles within units, and limitations in facility resources. Substantial opportunities exist for quality enhancement—by systematically improving EBP implementation rates, we can ultimately increase survival without major morbidity in this vulnerable population.

## Conclusions

Bundled implementation of EBPs significantly improves clinical outcomes in VPIs. However, suboptimal adherence—particularly for normothermia within the first hour after birth and DCC/UCM—necessitates targeted quality improvement initiatives. We can enhance quality and improve survival without major morbidity in VPIs by systematically improving the implementation rates of EBPs.

## Data Availability

The original contributions presented in the study are included in the article/Supplementary Material, further inquiries can be directed to the corresponding authors.
