# Supplementary material for: Association of individual and bundled evidence-based practices with adverse outcomes in very preterm infants: a multicenter retrospective study
Source: Front Pediatr. 2026 May 20;14:1785689. doi: 10.3389/fped.2026.1785689 (PMC13230218; doi:10.3389/fped.2026.1785689)
Supplement: Supplementary file 1 [file Table1.docx]

**Supplementary Table 1** Comparison of baseline characteristics between included and excluded very preterm infants

| **Variables, n(%)** | **Included**  **(n=536)** | **Excluded**  **(n=162)** | **χ^２^/Z** | **P value** |
| --- | --- | --- | --- | --- |
| Gestational Age(weeks)median (IQR) | 30(28.1,31) | 30.1(28.7,31.1) | -1.324 | 0.185 |
| Sex, Male | 313(58.4) | 76(46.9) | 6.647 | 0.01 |
| Birth Weight(g), median(IQR) | 1245(990,1490) | 1295(980,1492) | -0.44 | 0.66 |
| small for gestational age | 29(5.4) | 9(5.6) | 0.005 | 0.943 |
| ART | 109(20.4) | 19(11.9) | 5.965 | 0.015 |
| multiple pregnancy | 151(28.2) | 31(19.1) | 5.433 | 0.066 |
| maternal age(y), median(IQR) | 31(29,35) | 32(29,36) | -1.12 | 0.263 |
| Cesarean section | 371(69.6) | 122(71.3) | 0.173 | 0.677 |
| GDM | 166(31) | 53(32.9) | 0.205 | 0.651 |
| HDP | 108(20.3) | 39(24.1) | 1.082 | 0.298 |
| 1-minute Apgar score<7 | 99(18.5) | 33(20.4) | 0.293 | 0.588 |
| 5-minute Apgar score<7 | 20(3.7) | 7(5.2) | 0.617 | 0.432 |

Abbreviations: GDM, gestational diabetes mellitus; HDP, hypertensive disorders of pregnancy; ART, assisted reproductive technology.

* p<0.05 indicates statistical significance.
